# Characterizing the state of the art in the computational assignment of gene function: lessons from the first critical assessment of functional annotation (CAFA)

**DOI:** 10.1186/1471-2105-14-S3-S15

**Published:** 2013-04-22

**Authors:** Jesse Gillis, Paul Pavlidis

**Affiliations:** 1 Stanley Institute for Cognitive Genomic, Cold Spring Harbor Laboratory, 196 Genome Research Center, 500 Sunnyside Boulevard Woodbury, NY, 11797, USA; 2 Centre for High-Throughput Biology and Department of Psychiatry, University of British Columbia, 177 Michael Smith Laboratories 2185 East Mall, Vancouver, Canada, V6T1Z4

## Abstract

The assignment of gene function remains a difficult but important task in computational biology. The establishment of the first Critical Assessment of Functional Annotation (CAFA) was aimed at increasing progress in the field. We present an independent analysis of the results of CAFA, aimed at identifying challenges in assessment and at understanding trends in prediction performance. We found that well-accepted methods based on sequence similarity (i.e., BLAST) have a dominant effect. Many of the most informative predictions turned out to be either recovering existing knowledge about sequence similarity or were "post-dictions" already documented in the literature. These results indicate that deep challenges remain in even defining the task of function assignment, with a particular difficulty posed by the problem of defining function in a way that is not dependent on either flawed gold standards or the input data itself. In particular, we suggest that using the Gene Ontology (or other similar systematizations of function) as a gold standard is unlikely to be the way forward.

## Introduction

In computational biology, critical assessment of algorithms plays an important role in keeping the field honest about utility by ensuring progress is measurable and in a direction that is helpful in solving biological problems. The recognition of the need for assessment dates back to the first Critical Assessment of Structure Prediction (CASP), which aimed to determine the state of the art in protein structure prediction [1]. CASP's ongoing assessment has proven highly successful in characterizing progress, and 20 years later CASP largely defines the field of protein structure prediction. CASP has a number of features that are important to its success, some of which were built in from the start and others which were the result of lessons learned along the way. Among those features are forcing participants to make true predictions rather than blinded post-dictions (limiting over-training), the use of fully representative evaluation metrics (limiting artifactual performance), and the recognition of sub-problems that are treated as distinct tasks (allowing for different strategies, e.g. "template-free" vs. "template-based" prediction). In addition to these inherent lessons, CASP has taught the field that progress in "template-free" (*ab initio*) prediction, while substantial, is slower than prediction that can directly leverage existing protein structures thanks to sequence similarity. CASP's results have also shown that the aggregation of algorithms is an effective way to reduce the effect of "noisy" low-quality predictions [2].

CASP has inspired numerous other critical assessments, including the topic of this paper, the first Critical Assessment of Functional Annotation (CAFA). CAFA was aimed at assessing the ability of computational methods to predict gene function, starting from protein sequences. The general approach for predicting gene function is often referred to as "guilt by association" (GBA) [3]. In a computational GBA framework, the input data takes the form of similarities among genes (sometimes this is treated as a graph or gene network) and some initial functional labeling (often based on the Gene Ontology or a related scheme [4]). Genes which are in some sense "near" genes with a given label might be proposed to share that label (function) with them. While a common measure of "guilt" uses sequence similarity, numerous other data types have been used alone or in combination, such as coexpression, protein interactions, patterns of conservation and genetic interactions. All of these have been shown to be predictive of gene function to varying degrees when tested by cross-validation, and numerous algorithms of varying levels of sophistication have been proposed. However, independent assessment of computational GBA is not routine. CAFA represented an attempt to fill this gap. This is a very challenging goal due to problems in defining gold standards and evaluation metrics [5].

In the first CAFA, in which we were only observers, approximately 47000 protein sequences from UniProt were selected as targets. These sequences were chosen because they lacked high-quality evidence codes ("EXP", "TAS" or "IC") on any their Gene Ontology (GO) annotations (if they had any), and thus were considered to have "unknown function" (http://biofunctionprediction.org/node/262). Participants were asked to assign GO terms to the proteins, with no more than 1000 terms assigned to any given target. Importantly, the GO terms that would eventually be used for assessment were not known in advance, and users were left to decide which data to use as input to their algorithms, without restrictions. The large number of targets in CAFA and the unpredictable nature of which sequences would be used for assessment in the end ensured that it would be difficult for participants to "game" the system.

After participants submitted their predictions, a six month waiting period ensued. At the end of this period, UniProt/GOA was checked for updates to the GO annotations of the targets. New GO annotations which had "EXP", "TAS" or "IC" evidence codes were treated as the gold standard "truth" to which each participant's predictions would be compared. Such new GO annotations were available for ~750 sequences. As set out by the CAFA rules, performance for a submission was to be measured for each target, by comparing the GO annotation predicted by the algorithm with the truth. To capture the idea of "near misses", a novel measure of precision and recall were devised by the organizers using the number of common (up-propagated) GO terms shared by the truth and prediction.

CAFA was structured differently from an earlier assessment that had similar motivations, Mousefunc [6]. Mousefunc provided participants with a blinded set of prepared data (gene networks and the like), and an honor system was used to prevent the nine participating groups from reverse-engineering the coding. In addition to classifying a training set of genes against the target GO terms, participants made predictions for a held-out 10% of the genes, which were unmasked for the assessment. The conclusions of the Mousefunc assessors were that in general methods performed fairly similarly (combined yielding 41% precision at 20% recall), with one method standing out as the "winner" by a modest margin; and that "molecular function" terms were easier to predict than those for the "biological process" aspect of GO. By far the most informative data sets were those based on protein sequence (while sequences were not directly used, two of the data sets were founded on protein sequence patterns). A set of 36 novel predictions (that is, high-scoring apparent "false positives") were evaluated by hand and found to have literature support at an encouraging rate of around 60% [6]. Recently, we reanalyzed the Mousefunc data and many other gene networks and showed that much of the learnability of gene function in cross-validation is explained by node degree effects (to a first approximation, assigning all functions to "hubs" is a surprisingly effective strategy) [7]. We hypothesized that the problem of prediction specificity would play a similar role in CAFA results.

In this paper, we report the results of our independent assessment of a portion of the CAFA results made available to us. Our intention was to assist the CAFA organizers in making the most of the assessment, and to gain insight into how gene function predicts "in the wild". Our results suggest that most prediction algorithms struggle to beat BLAST. An evaluation based on an information-based metric suggest that informative predictions are made at a rate of at best 15%, and that many of the informative predictions made could be inferred from the pre-existing literature and GO annotations. In agreement with our previous results on multifunctionality, informative predictions tended to be made to GO groups containing highly multifunctional genes. We find a comparison of the CASP and CAFA tasks to be informative, in terms of the some lessons learned through CASP and how they might be applied to CAFA in the future. However, the evidence suggests that many of the challenges are fundamentally due to reliance on an imperfect gold standard.

## Methods

All data and methods we describe for CAFA were based on information that was publicly available to non-participants at the time of the assessment or shortly after the summer 2011 workshop. As noted in the discussion, there are a few points of variance in the assessment that was finally done by the organizers. Our methods reflect our understanding of the state of affairs as they would have appeared to a participant in the assessment.

### Data

The CAFA participants were contacted by the organizers at our behest, asking them if they would be willing to provide their predictions to us for analysis. It was made clear that we would be focusing on overall patterns and not on the performance of individual identified algorithms. Positive responses were received from 16 groups (no negative responses were obtained; the remainder were apparently non-responses). This yielded a set of results for 16 out of 56 algorithms that were entered in the assessment; for the sake of discussion we assume this subset is representative. In accordance with our agreement with the participants, in this paper we do not identify the specific algorithms or the participants. We were also not provided with any information about the input data used by the algorithms other than the target sequences that were provided. We note that it was straightforward for participants to determine existing annotations for the sequences, if they desired, so we assume this information was available for the purposes of our evaluation.

The format of the data we were given was as follows, for the Molecular Function (MF) and Biological Process (BP) categories separately. For each of up to 1000 GO terms per target, a score in the interval (0.00, 1.00] was provided, where 1.00 was to indicate the strongest prediction (some algorithms only provided binary scores). Non-predictions were indicated by missing values (that is, the value 0.00 was not allowed). Not all algorithms made predictions for all targets, and not all algorithms made the maximum of 1000 predictions for all targets. One submission did not provide predictions in the BP category. We only received predictions made for the evaluation targets and were thus unable to examine the results for the other ~46,000 targets.

In addition to the official entries, the organizers provided results of using BLAST [8] as a predictive method (assigning GO terms based on the top BLAST hit in the "nr" database, using default settings) and results from a BLAST-based method, GOtcha [9]. GOtcha takes into account information about the structure of the Gene Ontology in combining information from multiple high-scoring BLAST hits. Another data set was provided to us in which sequences were "predicted" to have GO terms according to the proportion of sequences which had a given term in the existing GO annotations. Thus all proteins were assigned the terms "Biological Process" and "Molecular Function" with weights 1.0, and terms "lower down" in the GO hierarchy with decreasing weights until 1000 GO terms were reached. We refer to this as the "Prevalence" data set. For reasons to be described the Prevalence data set is best considered a control, not a real entry; thus our evaluation focuses on 18 entries including BLAST and GOtcha. To create an aggregate classifier, the results from all 18 algorithms were combined using the average normalized rank of the scores provided for each algorithm. We chose this method of aggregation because it is as naïve an aggregation as we could imagine, involving no prior expectation as to performance or training based on data. We stress that it should not be regarded as a competitor algorithm since it merely averages the other algorithms' prediction data after the fact.

The annotations treated as a gold standard for evaluation involve 558 sequences in the biological process (BP) evaluation category, and 454 for molecular function (MF), from 10 difference taxa. These sequences were selected for evaluation from the starting set of 46997 because they had received GO annotations with "strong" evidence codes ("EXP", "TAS" or "IC") during the six month waiting period. After propagating these terms upwards in the GO hierarchy, a total of 2457 terms (out of 18982 possible) were annotated at least once in the BP category and 709 (out of 8728) in MP.

### Assessment metrics

The primary assessment metric proposed by the organizers is gene-centric (http://biofunctionprediction.org/node/262). For each gene, the terms assigned are propagated up the GO hierarchy to the root, yielding a set of terms. This is performed for each scored term a gene is given, starting with the highest scoring and working downward, adding to the number of predicted terms. This propagation could be done by the submitter, or would be done by the organizers for all non-zero predictions. The same propagation was done for the gold standard term. Any terms overlapping between these sets was considered "correct". Given these sets, precision and recall can be computed the usual way, (precision = |True terms|/|predicted terms|, recall = |correct predicted terms|/|True terms|). To generate a precision-recall curve, the predictions for each gene were treated as a ranked list of GO terms (with the non-assigned GO terms being tied at the bottom). When a term is expanded by up-propagation, the best score given for the term is the one retained (since submitters may have submitted an explicit score for a GO term as well as one or more scores implied by the structure of GO) unless the submitter had provided a specific lower score for that term. Precisions at each recall were computed, generating a precision-recall curve. The assessment rules did not specify a way in which to summarize these curves, so we used the average precision [10]. We refer to this as the "CAFA score".

We present results from two additional metrics. One is function-centric and is a standard area under the receiver operating characteristic curve (AUROC). We first propagated GO terms as described above. For each GO term in the gold standard, we considered it for ROC computation if it had 10-100 members. For each such GO term, across all evaluation targets, we used the scores provided by the submitter to rank the predictions, with non-predictions treated as tied. The area under the ROC curve was computed using standard methods. Because we did not get information on the unevaluated targets, the AUCs we computed are probably depressed, as we suspect that the prediction scores for the other ~46,000 would tend to be lower than that for the evaluation targets, due to biases in which of them received new GO annotations. However, this does not preclude valid internal comparisons based on the evaluation targets.

The second metric we used is gene-centric but attempts to overcome problems we encountered with the CAFA score. The measure is the semantic similarity between the actual and predicted function, using the measures of Resnik [11] or Lin [12]. These measures take into account the information content of each GO term, based on the usage of terms in existing annotations [13]. Thus getting a rarely-used GO term correct in a prediction is given higher weight than correctly guessing a generic one. We note that the use of these semantic similarity measures has been previously proposed for assessment of predictions [5].

### Gene Ontology annotations

To perform a retrospective analysis, we obtained annotations from the GOA [14] FTP archives, dated January 11 2011. For mouse this corresponds to GOA release 79; for human it is release 93; for E. coli release 91; rat release 79; Arabidopsis release 66. The deadline for CAFA submissions was January 18, 2011 so the file represents GO annotations that were available to the participants at the time of submission and could have in principle been used for inference. Note that our analysis is based on the presence of the annotations in the files for the date given, not the "date of annotation" column in the latest files; the latter appears to be misleading or incomplete.

### Algorithm aggregation

To combine the predictions into a single set, the gene rankings for each algorithm were normalized to the range 0-1. We computed the mean of this normalized rank for each gene, and ranked the resulting means to obtain the final ranking.

## Results

### Gene-centric evaluation using the CAFA score

**Figure 1:**
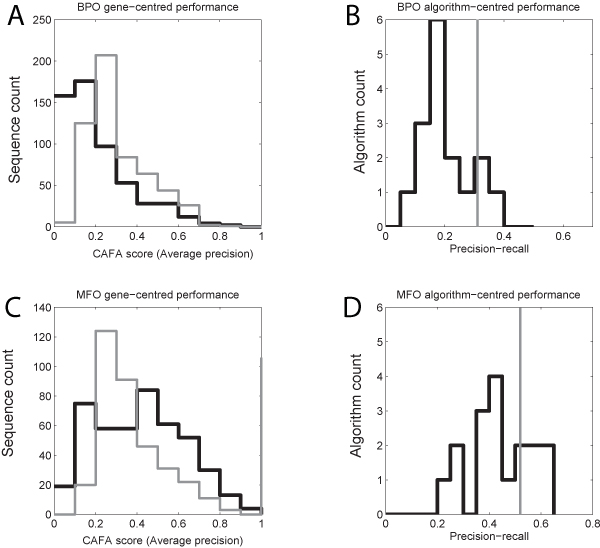
**Summaries of performance using the "precision-recall"-based CAFA score**. A and B show results for the BP ontology; C and D for MF. Submitted results are shown in black, and the null Prevalence data are represented in grey. A and C plot the distribution of scores for all evaluation targets, averaged across algorithms. B and D show the distribution of scores across algorithms, averaged across targets.

The 18 methods evaluated yielded mean CAFA scores of 0.45 for the Molecular Function ontology (MF) and 0.21 for Biological Process (BP) (Figure 1). The scores obtained by aggregating across all algorithms were better than any of the individual algorithms (0.40 for BP and 0.67 for MF). Whether these values are good in an absolute sense depends on what one considers the null distribution. If one completely ignores any prior knowledge of GO structure, the values appear to be very high. However, a more appropriate null is provided by the Prevalence data. Any algorithm that cannot do better than assignment of gene functions based on their existing prevalence among annotated genes is not useful in practice. The fact that the Prevalence method is not really a method but a "background" is highlighted by the fact that it gives the same predictions to all genes, and that it can be computed without any information about the target genes. Considering Prevalence as the baseline, only three algorithms perform better than null on BP, and six perform better than null on MF (Figure 1B and 1D). BLAST performed worse than the null (0.19 vs. 0.31 for BP, 0.44 vs. 0.52 for MF).

Further investigation revealed that this result is not due to terrible performance of the algorithms, but that the CAFA score is problematic. Consider an algorithm which simply predicts "Molecular function" (GO:0003674, the root of the MF hierarchy) for every target. This is always a correct (precise) prediction, because all MF evaluation targets are assigned the term "molecular function", due to the propagation of annotations to the root. One might hope this single correct prediction is counterbalanced by poor recall; however the recall turns out not to be that bad, because the evaluation targets for CAFA have few annotations, even after propagation (7.3 terms on average in MF). Thus simply assigning "Molecular function" to each target yields a respectable CAFA score of 0.19, which is clearly erroneous. The performance of the "Prevalence" data represents a generalization of this problem. In theory, the submitters could have compensated for this issue by making sure they had at least made the predictions they could make with little chance of decreasing their precision. If this was done in a consistent manner, the scores would at least be internally comparable, but there is no evidence to suggest the submitters took this into account. The implication of this analysis is that the CAFA score is not giving a useful representation of the performance of prediction algorithms in any absolute sense, and it also cannot be used to reliably compare the CAFA submissions to each other.

In prediction one can rank functions with respect to a gene ("Which functions are most associated with this gene?"), or one can rank genes with respect to a function ("Which genes are most associated with this function?"). The primary CAFA score is gene-centric. However, for a prediction to be useful it should be valid from either perspective. Otherwise one might be able to argue that all genes have all functions, in some weak or indirect sense. To make a useful functional assignment, we must consider whether a gene is "more involved" in a function than other genes. We therefore considered a more traditional "function's point of view" evaluation, and finally an alternative gene-centric evaluation that takes into account specificity of predictions and is thus indirectly comparative.

### Evaluation by area under ROC curves

A common way to evaluate predictions is to ask, for each function, which gene is most likely to possess that function. This is challenging to implement in the context of CAFA because of the capricious nature of which GO terms and genes were available for evaluation. For many GO terms there are simply too few genes which received a prediction. Selecting GO terms that had between 10 and 100 genes assigned to them yielded 245 terms for BP and 45 for MF. Because of the small number of targets overall, these tend to be somewhat "high level" GO groups.

**Figure 2:**
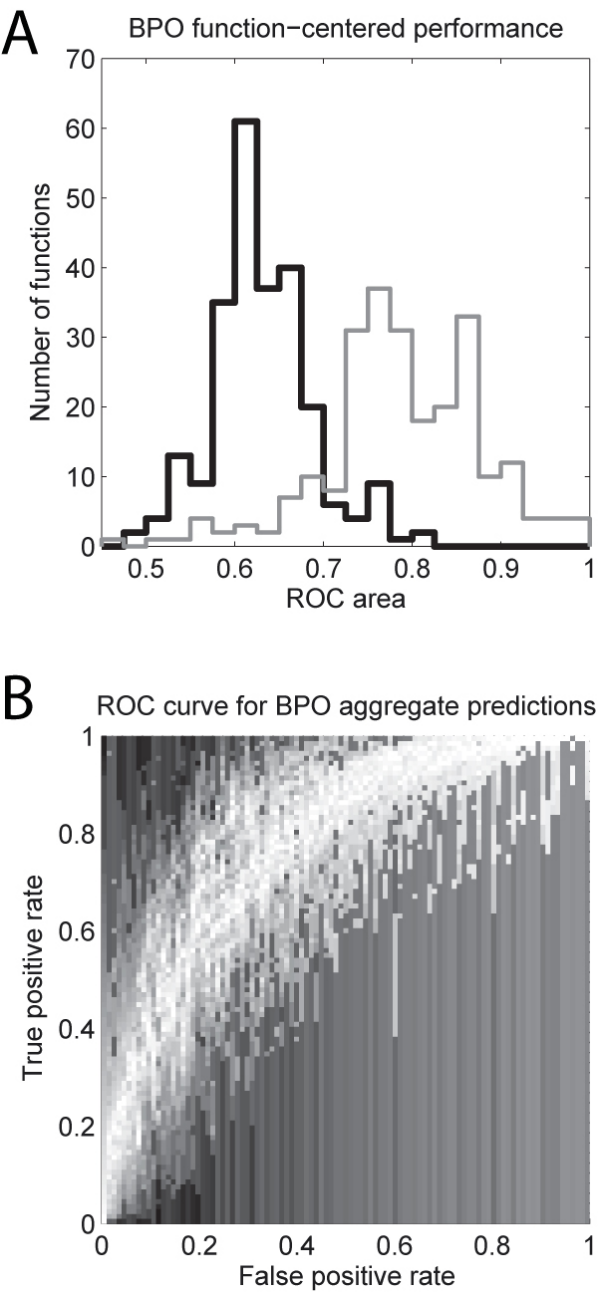
**Summaries of performance using ROC curves**. Results are only presented for BP because the MF results were too strongly affected by biases due to the *E. coli *annotations. A. Distribution of AUROCs for the GO terms evaluated. The mean performance across algorithms is shown in black. A simple aggregation algorithm does much better on average, shown in grey. B. Density plot showing the overlay of the ROC curves that make up the results shown for the aggregation algorithm in A, with areas of high density shown in lighter shades. Scattered light areas are artifacts due to the effects of GO groups with smaller numbers of genes. Note that the Prevalence method is guaranteed to generate AUROCs of 0.5 for all functions since it ranks all genes equally.

Switching to this function-centric perspective, the 18 algorithms now score quite well in BP with an average AUROC of 0.63 (Figure 2; note that the Prevalence data is guaranteed to score exactly 0.5 AUROC as it ranks all genes equally for each function). The best single method is BLAST, with a mean AUROC of 0.75. Once again, the aggregate outperforms the individual algorithms with a mean AUROC of 0.77. AUROCs for MF were also generally high, with the average performance being 0.66, BLAST scoring 0.71, and the aggregate performing better than any individual algorithm (0.84). We note that these values are likely to be somewhat artificially depressed, because they do not take account of the ~46,000 sequences that were left out of the evaluation due to still not being annotated with good evidence codes at the time of assessment; we expect that if the submitted scores for these were available, AUROC values would be higher (this assumes that the 46,000 would be biased towards sequences for which predictions would tend not to be made, due to lack of sequence similarity to annotated proteins, for example).

We also evaluated species-based variation in functional assignment, to account for the possibility of issues such as GO terms being used in only one species. For each GO term, sequences were ranked by the incidence of the term in the species from which the sequence was derived (that is, all sequences from a given species were given the same ranking). This yields a mean AUROC of 0.55 for BP (low, but significantly different from 0.5), while assessment per-species yielded AUROCs comparable to the overall results reported above. This suggests that species biases in term usage are not a major influence. However, the molecular function assignments were badly distorted by species-specific variation in assignments. In particular, *E. coli *assignments in MF were anomalous in having exceptionally high depth (large numbers of GO terms assigned per gene; Additional file 1. Terms for *E. coli *also had an unusually high correlation within the BP; Additional file 1). The reason for this is not clear, but may partially reflect the smaller number of annotations assigned to E. coli in the BPO relative to other species (17.6 vs. 30.5). However, this elevated annotation depth outside E. coli in BPO was extremely variable (standard deviation of 31.3), suggesting it cannot fully explain the species-wide correlation pattern. Curation practices in Ecocyc ([15], the source of *E. coli *GO annotations) may be a more important casual factor.

### Evaluation using information content

**Figure 3:**
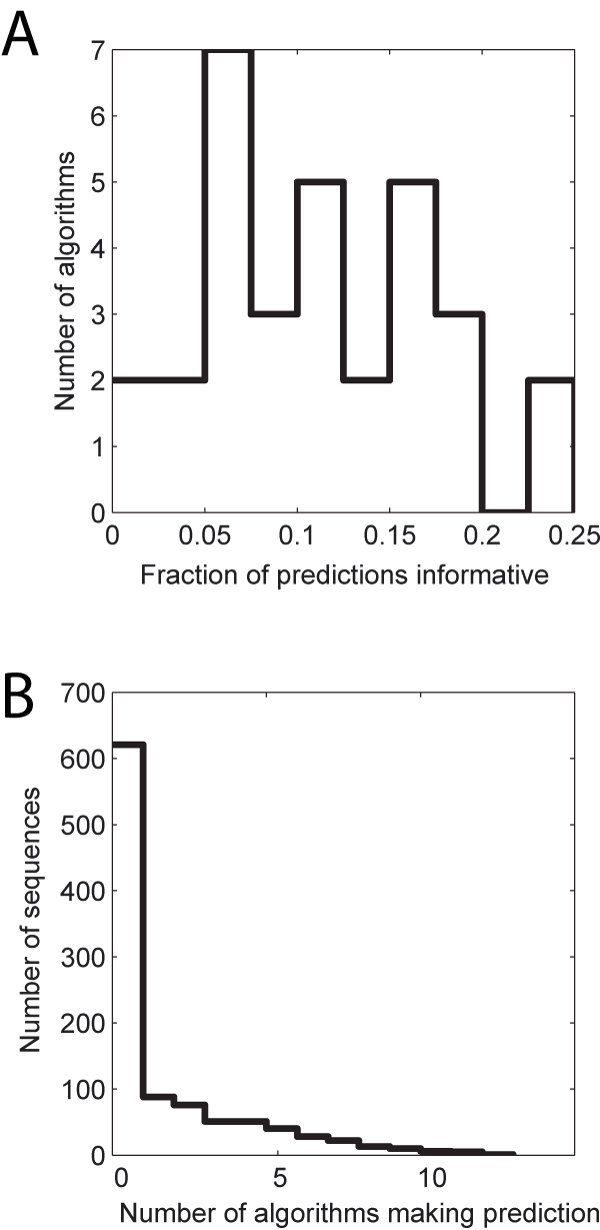
**Summaries of performance based on information content**. Results are only shown for BP because of the distorting effect of *E. coli *annotations in MF. A. The fraction of predictions considered informative per algorithm. B. Overlaps among informative predictions. Most sequences received no informative predictions (peak at 0), while numerous predictions are made by multiple algorithms.

The main problem with the CAFA gene-centred metric is its failure to account for the structure of GO in considering how informative a prediction is. We therefore considered a metric based on the semantic similarity between the hypothesized assignment and the true assignment. This allowed us to measure whether prediction algorithms ever assign a function which is "surprisingly" precise (unlikely to be made by chance). We quantified "surprise" by asking whether the most specific predictions are higher-scoring than the best scoring prediction made by the Prevalence method. Thus a novel prediction had to be more informative than the best "prediction" that could be made simply based on the null. All of the algorithms passed this threshold at least once within the BP category, and on average 7.6% of their predictions were more informative than the best prediction ever made by the Prevalence method (for any of the 558 genes), with a maximum of 14.7% (Figure 3A). BLAST yielded 5.6% while GOtcha yielded 9.3%. These findings were not substantially altered by choice of semantic similarity measure (Resnik or Lin) or different thresholds for which of a method's top N picks could be considered proposed functions. We obtained comparable results for the MF evaluation, with all algorithms generating at least some unusually informative predictions (again, relative to prevalence), with 10.0% percent of predictions being informative, on average. These results show that the algorithms can make correct and specific predictions, though at low proportions.

As shown in Figure 3B, algorithms often gave informative predictions for the same sequences. The majority of sequences had no strongly informative predictions made for them by any algorithm. While we do not know the methods or data sources used by the submitters (other than in the case of BLAST and GOtcha) the results suggest that these targets had some feature that made them especially predictable. The good performance of GOtcha led us to hypothesize that information on sequence similarity was responsible for these unusually good predictions.

To test the effect of sequence similarity we took advantage of the fact that many of the evaluation sequences already had GO terms prior to the assessment (in the BP, an average of 15 functions per sequence; we assessed this only for mouse and human). These annotations were present because it is routine for genes which have high sequence similarity to other better-annotated genes to be assigned GO terms with "weak" evidence codes such as "IEA", indicating a computational prediction. Over time, as experimental evidence is obtained, these evidence codes can be upgraded to those which were considered part of the CAFA evaluation (e.g., "TAS"). We tested whether using the pre-existing GO annotations as an entry to CAFA would have been competitive. Indeed, this yields performance tied with the best-performing algorithm in terms of the number of "informative" predictions (32 out of 275 in MF), with 16 due to the same sequences as the best-performing algorithm. This strongly suggests that in many cases, computational methods are simply recomputing the "IEA" annotations, and by happenstance some of those were upgraded in GO during the waiting period, making them part of the gold standard for the purposes of CAFA. We note that, presumably, the upgrading of the GO annotations is partly driven by the presence of the IEA annotations in the first place (or, at least, by the underlying sequence similarity); sequences which are already annotated in GO are also more likely to be experimentally tested, a form of confirmation bias. Thus these apparent "informative predictions" could be considered in part successful guessing of which sequences are likely to attract attention from experimentalists.

### Effect of gene multifunctionality

While we did not have access to the data used by the submitters, we wished to see if any underlying biases in the data could be ascertained by the behaviour of the algorithms. In particular we hypothesized that assignments would tend to be made to GO groups that contain multifunctional genes [7]. Indeed, functions populated more heavily by multifunctional genes were preferentially (rank correlation ~0.30) the function whose assignment caused a rise in the Lin semantic similarity (which, unlike Resnik similarity, is sensitive to false positives).

### Manual examination of informative biological process predictions

To gain further insight into how predictions are made, we more closely examined some of the "most informative" predictions (the top ten such predictions for BP from the aggregated algorithms are listed in Table 1). We used GO annotations from before the start of CAFA (early January 2011) and compared them to the annotations that appeared during the waiting period. This analysis was assisted by the UniProtKB [16] and QuickGO [17] web sites but relied primarily on the annotation files provided by GOA [14].

**Table 1:** The sequences with the top ten "most informatively" predicted correct annotations by the aggregate algorithm are summarized.

Sequence	Gene symbol	Gold standard	Closest informative prediction	Pre-existing IEA terms (representative)	Pub Date
BHMT1_MOUSE	Bhmt	methionine biosynthetic process (GO:0009086)	methionine biosynthetic process (GO:0009086)	methionine biosynthetic process	2004

IPO13_RAT	Ipo13	steroid hormone receptor nuclear translocation (GO:0002146)	protein import into nucleus, translocation (GO:0000060)	protein import into nucleus	2006

ARGB_ECOLI	argb	arginine biosynthetic process (GO:0006526)	arginine biosynthetic process (GO:0006526)	arginine biosynthetic process	2007

CAF1K_ARATH	CAF1-11	nuclear-transcribed mRNA poly(A) tail shortening (GO:0000289)	nuclear-transcribed mRNA poly(A) tail shortening (GO:0000289)	poly(A)-specific ribonuclease activity	2009

CFAB_MOUSE	Cfb	complement activation, alternative pathway (GO:0006957)	complement activation, alternative pathway (GO:0006957)	complement activation	1983

CHM4B_HUMAN	CHMP4B	endosome transport (GO:0016197)	endosome transport (GO:0016197)	protein transport; late endosome membrane	2010 (Reactome)

HA15_MOUSE	H2-T23	antigen processing and presentation of endogenous peptide antigen via MHC class Ib via ER pathway (GO:0002488)	antigen processing and presentation of endogenous peptide antigen via MHC class Ib (GO:0002476)	antigen processing and presentation of peptide antigen via MHC class I	1992

SOX11_MOUSE	Sox11	positive regulation of hippo signaling pathway (GO:0035332)	embryonic digestive tract morphogenesis (GO:0048557)	cell differentiation;nervous system development	2010

TGT_ECOLI	tgt	tRNA wobble guanine modification (GO:0002099)	queuosine metabolic process (GO:0046116)	queuine tRNA-ribosyltransferase activity	1982

VATL_MOUSE	Atp6v0c	lysosomal lumen acidification (GO:0007042)	lysosomal lumen acidification (GO:0007042)	proton-transporting V-type ATPase, V0 domain;vacuole	2001

The date of publication of the gold standard source is given in the last column.

As shown in Table 1, in seven of the top 10 cases, the aggregate algorithm included the "right answer" among the predictions meeting the threshold established by the Prevalence baseline (although it was never the top prediction, not shown). Table 1 shows that in nearly every case, very closely related GO terms were already present before CAFA, in agreement with the systematic analysis described above. A possible exception is SOX11. Note that these "similar" terms might not be in the BP ontology, but because of correlations among the GO hierarchies, such terms are likely to be informative (for example, the cellular components "vacuole" for Atp6v0c and "late endosomal membrane" for CHMP4B). In all ten cases, the source of the annotation was available before CAFA, in some cases decades before (last column of Table 1).

## Discussion

CAFA provided a unique opportunity to evaluate computational gene function assignments. Our results provide some new insights into the behaviour of gene function prediction methods, and into the challenges in providing an adequate and fair evaluation. Some of these challenges have been noted prospectively [5] so it is interesting to see how practice meets theory. We focus to some extent on comparing CAFA to CASP, and where helpful lessons could be learned.

### Task categorization

One area where CAFA could follow CASP is in the definition of tasks. Currently CASP differentiates between three categories of tasks, all of which have direct analogies with function prediction tasks.

The CASP "template-based" prediction task is analogous to the case of trying to predict function when the gene has sequence similarity to already functionally annotated genes. In such cases, methods like BLAST provide a baseline for what can be learned readily. Our analysis shows that many of the CAFA targets already had "IEA" functions assigned, and to an extent CAFA successes are simply recovering these. Thus perhaps unsurprisingly, BLAST did well in the part of CAFA we had access to, and we expect that other high-scoring methods are using sequence similarity information. Tasks which exploit sequence similarity should be considered a distinct category of function prediction problems. Similarly, the CASP "template-free" prediction task is akin to the task of predicting gene function when no sequence similarity information is available (or at least, not used).

The CASP "structure refinement" task [18] might be analogous to the task of "function refinement" where an already functionally annotated gene is given new or more specific functions. We believe this could be treated as is a different task from assigning functions to a completely unannotated "orphan" gene (not having even IEA annotations). Among methods that fall into this category are those which use GO itself as a measure of "guilt" [19,20]. Thus if two genes share nine out of ten GO terms, the tenth one is a pretty good bet. Even if they don't explicitly rely on existing annotations, algorithms that are good at "refinement" might not be very good at "template-based" assignment (and vice versa).

We propose that some scheme like this be adopted for future CAFA assessments, to more clearly differentiate between cases where sequence similarity is highly informative and those where it is not, and possibly to extend the competition to include targets which already have some functions assigned with "strong" evidence codes.

### The importance of evaluation metrics

Over the years, CASP has modified its assessment metrics and now has an agreed-upon set of metrics. As we have shown, the primary performance metric initially proposed for CAFA is unsatisfactory. This is illustrated by the fact that by this measure, a null "prediction method" outperforms most methods. The problem with the CAFA score is that it is not comparative across genes. When one is predicting a function for a gene, the goal is to say that "this gene has function × more than other genes do" in some sense. Otherwise, the definition of function becomes degenerate, and simply assigning all genes the same functions becomes reasonable.

We have applied two alternative measures, one which is gene-centric and focuses on the information content of a prediction, and a standard metric (AUROC) which is function-centric. The information-based metric is implicitly comparative, because it uses information on the distribution of GO terms across genes as well as a threshold set by the null predictor. The AUROC metric also ranks genes against each other. By these measures, it can be seen that the prediction algorithms (including BLAST) are providing meaningful performance. The problem with the function-centric measure is that it depends on having more than one prediction for the function to be scored, which cannot be guaranteed given the nature of the CAFA task. The differences among annotation practices for different organisms (notably for *E. coli *in the current data) make assessment even harder, as criteria vary for what is considered good annotations.

### The power of aggregation

In recent years, the top algorithms for CASP have tended to be meta-algorithms which aggregate the results of individual methods. If our experience is representative, the same is likely to be true for CAFA. The aggregate algorithm outperforms all the individual algorithms. The reason for this is apparently because aggregation allows a few "confident" predictions to rise to the top, while less confident predictions (which turn out to be poor) are "averaged out". A similar phenomenon was reported in the DREAM5 assessment of gene network inference [21].

### The benefit of having a clear goal

The points raised thus far are predicated on the idea that function prediction is like protein structure prediction. However, in a fundamental way this is not the case, at least not yet. Algorithms that perform well in CASP are considered to do well at "structure prediction". That is, the CASP tasks are well-aligned with what the field agrees the "real life" task is. This is basically because protein structure is fairly easy to define (position of atoms in space). In contrast, "gene function" does not have an agreed-upon definition. Certainly there is no consensus that the Gene Ontology is even close to biological reality, rather than just being convenient. Since function assignment/prediction methods always use experimental data as inputs, there may be more value in simply trusting those data than in trying to "align" predictions to a gold standard that is acknowledged by its creators to be problematic for such uses [22]. Tuning algorithms to be good at predicting GO annotations is probably never going to be satisfying. The task of interest is predicting gene function, not predicting GO annotations as an end in itself, and the fact that these two tasks are not congruent presents a challenge.

CASP also differs from CAFA in having targets defined by specific experimental results held back from participants, who are given key underlying data to use for computational analysis. The same approach is taken in the Critical Assessment of Genome Interpretation (CAGI, https://genomeinterpretation.org/). This has the benefit of anchoring the assessment in a very specific outcome measure (that is: did the computation agree with the experiment). However, it limits the number and scope of tasks that can be assessed. This model is not likely to be applicable to the general problem of gene function assignment, but might be useful as a way to make progress on more specialized problems.

It is worth mentioning that there are "function prediction" tasks that are not based on GO (or similar schemes) in the same way as CAFA. For example, some groups attempt to predict mutant phenotypes [19] (some of the CAGI tasks are of this type). The roles of the issues we raise in such situations are not entirely clear, but we note that the types of data used are the same as those used in the CAFA-style annotation task, and GO often figures prominently in such work, especially as a source of validation [23].

### Predicting evidence codes and "post-diction"

With the caveat that CAFA's evaluation is based on a relatively small number of proteins, and our analysis on a subset of the CAFA entries, there are some important themes that emerged in terms of which informative predictions were made. The evidence strongly suggests that a major factor is the availability of sequence similarity information. Finding a set of proteins which are not annotated at all was difficult, so many of the evaluation targets already had "IEA" annotations (presumably often based on BLAST or a similar approach). The successful predictions are in part simply guessing that those existing annotations are likely to be supported by experimental evidence once they are tested, and thus upgraded in GO. The strong influence of sequence similarity was also suggested by the Mousefunc study [6].

The fact that many of the most predictable annotations were based on literature reports that predate CAFA further suggests that a bottleneck in filling in GO is information retrieval from the literature, not prediction *per se*. Strictly speaking, many of the CAFA evaluation targets are "post-dictions". The short time window available to CAFA probably helped ensure this would be a factor; there was little chance that many experimental reports would be published and also curated in a six month period. The organizers were aware of this, and it is unlikely that CAFA participants would have been able to efficiently exploit the existence of publications describing the functions of proteins in the target set. On the other hand, for all we know some of the entries may have used text mining methods as a tool for making predictions (see Addendum, below). This might be considered yet another category of automated annotation task. But we stress that all the predictions are based on experimental data of one type or another, so this distinction may not be helpful.

This returns us to the issue of the relationship between function prediction and GO. If computational predictions are based on experimental data that could be used by curators to populate GO, then the task of prediction is reduced to simply copying that information into GO (with appropriate evidence codes), rather than considering GO to be some independent entity that algorithms should attempt to match.

## Conclusions

Our analysis is based on a subset of a single assessment of automated annotation of a relatively small number of proteins, but some general principles and concerns emerged which are very likely to be relevant to any assessment of function assignment. Sequence similarity appears to be a dominant influence on the ability to prediction function. In terms of performing assessment, clearly a major challenge is the relatively slow pace and unpredictable nature of the entry of experimentally-defined functions into the annotation databases. But perhaps the deepest issue is the difficulty of deciding what it means to predict function in a useful way, as the current gold standards are deeply problematic. The first CAFA was a bold attempt to put gene function prediction on a firmer footing, and we expect that future iterations will continue to promote progress in this difficult area of computational biology.

## Addendum

Since our manuscript was submitted and revised, a detailed paper describing the outcome of the CAFA evaluation appeared [24], which enhances the interpretation of our analysis. First, it is now clear that our assessment included one of the top performing methods, Argot2 [25], as finally judged by the organizers, lending weight to our reassessment as a fair representation of the entries. Second, it is reported that the organizers of CAFA did not evaluate the results using the precise rules which were released before the assessment (those which were available to us as non-participants). For example, target annotations for a specific term ("protein binding") were "not considered informative" and thus excluded from the main evaluation [24]; including this term pushes the naïve Prevalence score to be among the top performers (Supplementary Figure 3 of [24]). The organizers also interpreted "pick the highest scoring term among all non-zero predictions and propagate it to the root of the ontology" as excluding the root itself for evaluation (this has a minimal effect on the value of prevalence since it is merely the exemplary case of a general problem). Finally, the number of evaluation targets reported in [24] varies from ours apparently because the set was expanded after the initial assessment.

The most striking distinction between Radivojac et al. and our results, at first glance, is that we found that simple methods relying on sequence similarity were highly competitive, while Radivojac et al. ranked BLAST poorly stating that, "top algorithms are useful and outperform BLAST considerably," and, "BLAST is largely ineffective at predicting functional terms related to the Biological Process ontology" [24]. This conclusion was apparently based on the CAFA score; the authors did not report per-algorithm (function-oriented) AUROCs, on which basis BLAST ranks highest in our analysis. The issue is readily resolved by stressing that based on the CAFA score, the Prevalence score outperforms BLAST (sometimes even after removing "protein binding") and indeed other more sophisticated methods [24].

In any case it is clear that sequence similarity was the bedrock of function prediction in CAFA. As noted by Radivojac et al., nearly of the methods submitted use sequence similarity. All of the top performers use such methods, and for some (e.g., Argot2) it was the primary or sole source of data (not counting the use of GO itself). The overall top-scoring group ("Jones-UCL") reported that "the largest contribution to correct predictions came from homology-based function prediction" (supplement of [24]). The only useful non-sequence source of information cited for the Jones-UCL method was text-mining of UniProt-presumably amounting to post-dictions of the type we report in Table 1. The Jones-UCL method also took into "account the GO ontology structure to produce final GO results". Argot2 also leverages the structure of GO, using information related to the Prevalence score [25]. This reinforces our concern that tuning algorithms to match the assessment, while beneficial in a rarified sense, could be misleading about generalization to many real applications (see also [26]).

## Competing interests

The authors declare that they have no competing interests.

## Authors' contributions

Experiments were designed by JG and PP. JG performed most of the analysis and prepared the figures. PP drafted the manuscript with input from JG.

## Funding

Supported by NIH Grant GM076990, salary awards to PP from the Michael Smith Foundation for Health Research and the Canadian Institutes for Health Research, and a postdoctoral fellowship to JG from the Michael Smith Foundation for Health Research.

## Supplementary Material

Additional file 1 **Taxon-specific effects on annotation**. The GO annotations used as evaluation targets were used (not predictions). For each sequence, a binary vector of GO annotations was created (1= sequence is annotated), and the correlation among these vectors is plotted, with lighter shades indicating high correlations. The sequences are organized by taxon, with the E. coli sequences indicated. It is evident that the E. coli sequences have very high correlations in their annotations in BP (A), very low correlations in MF (B) and consistently high depth (number of terms assigned per sequence within the MFO; C). Depth of coverage exhibits no visually clear trend for E. coli within BPO, but is significantly depressed relative to other species (p<10^-6^, ranksum test). Click here for file
