# Supplementary figures and images for: Characterizing the state of the art in the computational assignment of gene function: lessons from the first critical assessment of functional annotation (CAFA)
Source: BMC Bioinformatics. 2013 Apr 22;14(Suppl 3):S15. doi: 10.1186/1471-2105-14-S3-S15 (PMC3633048; doi:10.1186/1471-2105-14-S3-S15)

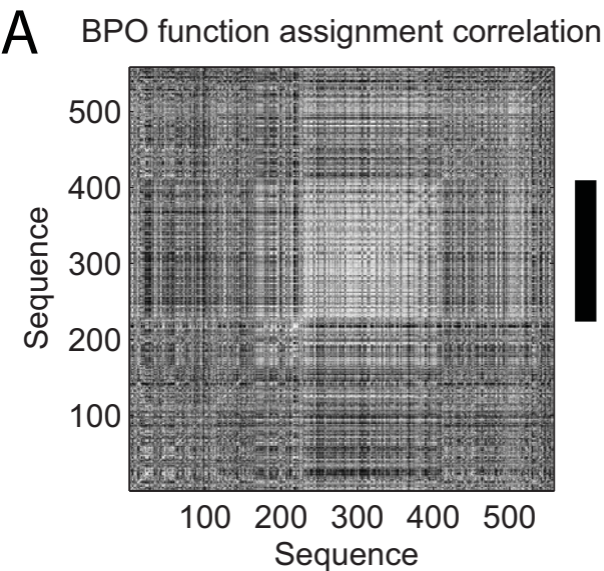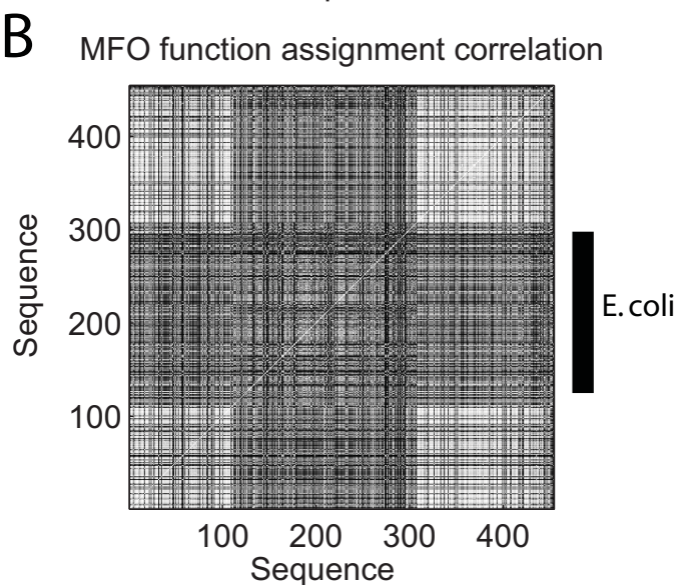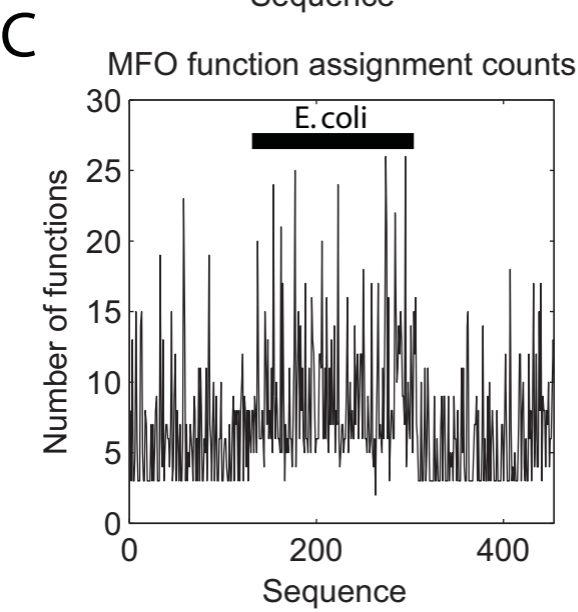

Supplement: Additional file 1 — Taxon-specific effects on annotation. The GO annotations used as evaluation targets were used (not predictions). For each sequence, a binary vector of GO annotations was created (1= sequence is annotated), and the correlation among these vectors is plotted, with lighter shades indicating high correlations. The sequences are organized by taxon, with the E. coli sequences indicated. It is evident that the E. coli sequences have very high correlations in their annotations in BP (A), very low correlations in MF (B) and consistently high depth (number of terms assigned per sequence within the MFO; C). Depth of coverage exhibits no visually clear trend for E. coli within BPO, but is significantly depressed relative to other species (p<10-6, ranksum test). [file 1471-2105-14-S3-S15-S1.PDF]
